# Mechanism of a Volatile Organic Compound (6-Methyl-2-Heptanone) Emitted From *Bacillus subtilis* ZD01 Against *Alternaria solani* in Potato

**DOI:** 10.3389/fmicb.2021.808337

**Published:** 2022-01-13

**Authors:** Dai Zhang, Ran Qiang, Jing Zhao, Jinglin Zhang, Jianing Cheng, Dongmei Zhao, Yaning Fan, Zhihui Yang, Jiehua Zhu

**Affiliations:** ^1^College of Plant Protection, Hebei Agricultural University, Baoding, China; ^2^Beijing Laboratory for Food Quality and Safety, Beijing Technology and Business University, Beijing, China; ^3^Agricultural Business Training and Entrepreneurship Center, Hebei Agricultural University, Baoding, China

**Keywords:** *Alternaria solani*, *Bacillus subtilis*, 6-methyl-2-heptanone, antifungal activity, conidial genes

## Abstract

The antagonistic mechanisms of soluble non-volatile bioactive compounds, such as proteins and lipopeptides emitted from *Bacillus* have been widely studied. However, there are limited studies on the antifungal mechanisms of volatile organic compounds (VOCs) produced by *Bacillus* against plant fungal diseases. In this study, the antagonistic mechanisms of one specific VOC, 6-methyl-2-heptanone, against *Alternaria solani* were investigated. To optimize the extraction conditions of headspace solid-phase microextraction, a 50/30-μm divinylbenzene/carboxen/polydimethylsiloxane fiber at 50°C for 40 min was used. For gas chromatography-mass spectrometry using a free fatty acid phase capillary column, 6-methyl-2-heptanone accounted for the highest content, at 22.27%, of the total VOCs from *Bacillus subtilis* ZD01, which inhibited *A. solani* mycelial growth strongly *in vitro*. Therefore, 6-methyl-2-heptanone was selected as the main active chemical to elucidate the action mechanisms against *A. solani*. Scanning and transmission electron microscopy analyses revealed that after exposure to an EC_50_ dose of 6-methyl-2-heptanone, *A. solani* hyphal cells had a wide range of abnormalities. 6-Methyl-2-heptanone also caused the capture of cellular fluorescent green label and the release of adenosine triphosphate (ATP) from outer membranes *A. solani* cells, which may enhance 6-methyl-2-heptanone ability to reach the cytoplasmic membrane. In addition, 6-methyl-2-heptanone showed strong inhibitory effect on *A. solani* conidial germination. It also damaged conidial internal structures, with the treated group having collapsed shrunken small vesicles as observed by transmission electron microscopy. Because 6-methyl-2-heptanone showed strong effects on mycelial integrity and conidial structure, the expression levels of related pathogenic genes in *A. solani* treated with 6-methyl-2-heptanone were investigated. The qRT-PCR results showed that transcriptional expression levels of *slt2* and *wetA* genes were strongly down-regulated after exposure to 6-methyl-2-heptanone. Finally, because identifying the functions of pathogenic genes will be important for the biological control of *A. solani*, the *wetA* gene was identified as a conidia-associated gene that plays roles in regulating sporulation yield and conidial maturation. These findings provide further insights into the mechanisms of VOCs secreted by *Bacillus* against *A. solani*.

## Introduction

Potato early blight caused by *Alternaria solani* is a main factor in the death of potato leaves, and it results in substantial yield losses (Morgan et al., 2002; Pasche et al., 2007; Peters et al., 2008). Fungicides are the main effective methods of controlling potato early blight disease. However, because of increased fungal pathogen drug resistance, environmental pollution and human health risks induced by the abuse of chemical fungicides (Wang and Liu, 2007; Maachia et al., 2015), there is a greater need for alternative environmentally friendly effective methods to control fungal diseases of potato.

Biological control has been widely regarded as a potential substitute for chemical fungicides owing to its environmental safety and high efficiency. The use of *Bacillus* strains as the biocontrol microorganisms is presently a promising strategy for controlling plant pathogens (Chaurasia et al., 2005; Zheng et al., 2013). *Bacillus* strains exhibit significant antifungal activities against various pathogenic fungi, such as *Penicillium digitatum*, *Monilinia fructicola*, and *Botrytis cinerea* (Wichitra et al., 2008; Senthil et al., 2011; Banani et al., 2015; Maachia et al., 2015; Liu et al., 2018). Recently, the use of volatile organic compounds (VOCs) produced by *Bacillus* strains was proposed as an alternative control method for plant fungal diseases (Chen et al., 2008; Gao et al., 2017; Haiyan et al., 2018; Massawe et al., 2018; Zhang X. Y. et al., 2020) because of their strong inhibitory effects on plant fungi (Chaves-López et al., 2015; Raza et al., 2016a,b; Gotor-Vila et al., 2017; Calvo et al., 2020). In addition, various VOCs produced by *Bacillus* strains have been identified as effective compounds (e.g., 2-nonanone, 2-methylpyrazine, and benzothiazole) (Arrebola et al., 2010; Raza et al., 2015; Khan et al., 2018; Liu et al., 2018; Xie et al., 2018; Wu et al., 2019; Calvo et al., 2020).

Recent research on the VOCs produced by *Bacillus* strains has primarily focused on evaluating volatile mixture biocontrol effects, including disease incidences in inoculated plants, spore germination, mycelial growth inhibition, and reduced sporulation, as well as the identification of exact VOC components (Kai et al., 2009; Schreinemachers and Tipraqsa, 2012; Khan et al., 2018; Tran et al., 2020). However, little is known about the biocontrol effects of specific identified VOC compounds. Moreover, the VOC types secreted by *Bacillus* strains are varied, including aldehydes, ketones, alcohols, and esters (Morath et al., 2012; Chantal et al., 2014). Additionally, different volatile chemicals do not have the same effects on, or the same degree of inhibition against, all fungi. This may be because different fungi respond to different component(s) of the volatile mixture and have different sites of action (Kai et al., 2009). For example, 3-methyl-1-butanol inhibits the mycelial growth of *Fusarium oxysporum* f. sp. *lactucae* but has no antifungal activity against *A. alternata* and *B. cinerea* (Chaves-López et al., 2015). Therefore, it is essential to study the biocontrol functions of *Bacillus*-specific volatiles to further determine the action modes of VOCs secreted by *Bacillus* on fungal pathogens.

In our previous study, VOCs secreted by *B*. *subtilis* ZD01 exhibited significant antifungal activity against *A. solani* (Zhang D. et al., 2020). In this study, we discovered that 6-methyl-2-heptanone was the dominant component in the VOCs, and it also shows strong antagonistic effects against *A. solani* (Zhang D. et al., 2020). Therefore, we speculated that 6-methyl-2-heptanone plays the most important role in pathogen inhibition. Consequently, we investigated the inhibitory effects of 6-methyl-2-heptanone produced by *B*. *subtilis* strain ZD01 on *A. solani*. Moreover, we identified the function of the *wetA* gene in *A. solani*. The results increase our knowledge of bacterial and fungal interactions mediated by VOCs and provide a potential strategy for potato early blight disease control.

## Results

### Optimization of Extraction Conditions and Identification of Volatile Organic Compounds Produced by *B. subtilis* ZD01

Different extraction conditions for headspace solid-phase microextraction (HS-SPME) affect the extracting efficiency. Moreover, columns with different polarities also affect gas chromatography-mass spectrometry (GC-MS) analyses. In this study, the effects of extraction fibers, time, and temperature conditions on the HS-SPME of VOCs produced by *B. subtilis* ZD01 were evaluated, and a free fatty acid phase (FFAP) capillary column (60 m × 0.25 mm × 0.25 μm) was used for GC-MS analyses of the produced volatiles. The total number of compounds and peak areas of the VOCs were used as the evaluation index.

**FIGURE 1:**
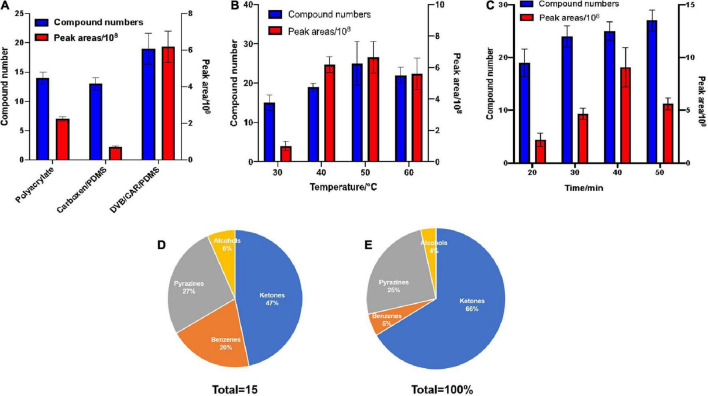
Optimization of collection conditions with HP-SPME for the GC-MS determination of VOCs. The effects of extraction fibers, time, and temperature on HS-SPME results for VOCs produced by *B. subtilis* ZD01 were evaluated. Total number of compounds and peak areas of VOCs served as the evaluation index. **(A)** Extraction fibers. **(B)** Extraction temperature. **(C)** Extraction time. After extraction, the analytes were identified by GC–MS. The compounds were classified into different classes based on their structures and functional groups, and we calculated the total peak areas of each of the four classes. **(D)** Classification of VOCs produced by ZD01. **(E)** Peak areas of four identified classes of VOCs from ZD01. The results are presented as the means ± SDs (*n* = 3).

Three kinds of extraction fibers [85-μm polyacrylate, 50/30-μm divinylbenzene/carboxen/polydimethylsiloxane (DVB/CAR/PDMS) and 75-μm CAR/PDMS] were tested. The 85-μm polyacrylate, 75-μm CAR/PDMS, and 50/30-μm DVB/CAR/PDMS fibers allowed GC-MS resolutions of 14, 13, and 19 distinct VOCs, respectively. In addition, a larger total peak area for the VOCs was obtained using 50/30-μm DVB/CAR/PDMS compared with the other extraction fibers (Figure 1A). Thus, the use of 50/30-μm DVB/CAR/PDMS increased the number and contents of VOCs. Consequently, we chose 50/30-μm DVB/CAR/PDMS as the extraction fiber for the following experiments.

The effect of extraction temperature on the GC-MS characterization of VOCs was analyzed. As shown in Figure 1B, 15, 19, 25, and 22, total compounds were detected by GC-MS at extraction temperatures of 30, 40, 50, and 60°C, respectively. In addition, the peak area of the total compounds was greatest at 50°C. Compared with other extraction temperatures, at 50°C the numbers and peak area of the total compounds detected by GC-MS were increased obviously. Thus, 50°C was selected as the extraction temperature.

We also evaluated the potential influence of extraction time on the GC-MS analysis of VOCs using 50/30-μm DVB/CAR/PDMS at 50°C. The extraction times of 20, 30, 40, and 50 min allowed GC-MS identification of 19, 24, 25, and 27 VOCs, respectively. In addition, the peak area of the total compounds was maximum at 40 min, which was much greater than at the other time points (Figure 1C). Therefore, we selected to use the 50/30-μm DVB/CAR/PDMS fiber at 50°C for 40 min as the extraction conditions for GC-MS.

Gas chromatography-mass spectrometry with a FFAP capillary column (60 m × 0.25 mm × 0.25 μm) was used to detect the volatiles of ZD01 samples and control samples (Luria-Bertani medium alone) under optimized extraction conditions (50/30 μm DVB/CAR/PDMS fiber at 50°C with 40 min). LB medium without *Bacillus* inoculation was used as a control. Identical volatile compounds produced by ZD01 and LB medium were eliminated. In total, 15 volatiles specifically released by ZD01 were obtained, including 7 ketones, 4 pyrazines, 3 benzenes, and 1 alcohol (Figures 1D,E and Table 1). Among them, 6-methyl-2-heptanone showed the largest peak area, at 22.27% of the total VOCs. Moreover, 6-methyl-2-heptanone was identified as having an 8.88% peak area of the total VOCs by GC-MS using an HP-5 capillary column and has shown strong antagonistic effects on *A*. *solani* (Zhang D. et al., 2020). Therefore, 6-methyl-2-heptanone may be the main active chemical; therefore, it was selected as a potential agent for controlling potato early blight.

**TABLE 1:** Volatile compounds produced by *Bacillus subtilis* ZD01 identified under optimal conditions using an free fatty acid phase (FFAP) chromatographic column.

No.	Chemicals	CAS	Retaining time (min)	SI	RSI	Peak area ratio (%)
1	3-Methyl-2-pentanone	565-61-7	7.27	863	913	3.32
2	2-Heptanone	110-43-0	10.22	867	877	4.42
3	6-Methyl-2-heptanone	928-68-7	11.46	888	892	22.27
4	5-Methyl-2-heptanone	18217-12-4	11.90	855	858	19.08
5	Methyl-pyrazine	109-08-0	12.41	939	941	6.65
6	2,5-Dimethylpyrazine	123-32-0	13.73	944	946	12.47
7	6-Methyl-2-heptanol	4730-22-7	14.58	874	885	3.53
8	2-Ethyl-5-methyl-pyrazine	13360-64-0	15.20	687	826	3.87
9	2-Methyl-5-(1-methylethyl)pyrazine	13925-05-8	15.81	845	975	2.00
10	2-Decanone	693-54-9	16.59	821	871	10.77
11	Acetophenone	98-86-2	21.90	639	863	4.70
12	2-Ethyl-6-methyl-phenol	1687-64-5	26.59	693	871	3.24
13	Phenylethyl alcohol	60-12-8	27.44	688	882	1.34
14	1,2-Benzisothiazole	272-16-2	28.63	772	922	0.53
15	2-Nonadecanone	629-66-3	30.45	731	869	1.81

*SI, strength indexes; RSI, relative strength indexes.*

### 6-Methyl-2-Heptanone Inhibited Mycelial Growth and Induced Structural Changes in *A. solani in vitro*

**FIGURE 2:**
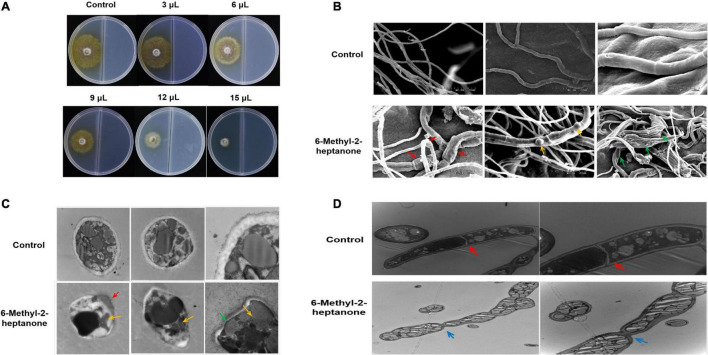
Mycelial growth of *A. solani* after the 6-methyl-2-heptanone treatment *in vitro*. A 5-mm square plug of an *A. solani* mycelial agar disc was placed in one compartment of the divided plate containing PDA medium, and the other compartment containing PDA medium was incubated with different aliquots of pure 6-methyl-2-heptanone. **(A)** Antagonistic effects of five different doses (3, 6, 9, 12, and 15 μL) of 6-methyl-2-heptanone against *A. solani* mycelia. **(B)** Scanning electron micrographs of *A. solani* co-cultured with 6-methyl-2-heptanone. The “red arrows” represented wrinkled surface cells of hyphae treated with 6-methyl-2-heptanone; the “yellow arrows” represented swelling tissues on the surface of hyphae after exposure to 6-methyl-2-heptanone; “green arrows” represented 6-methyl-2-heptanone-treated mycelia deformed and significantly enlarged. **(C, D)**. Transmission electron micrographs of *A. solani* hyphae co-cultured with 6-methyl-2-heptanone. The “green arrows” represented thinner cell walls; “red arrows” represented the movement of the cytoplasmic content towards the ruptured cell walls or cytoplasmic membranes; “yellow arrows” represented shrunken cytoplasm and decreased number of inclusions in Figure 2C. The “red arrows” represented clear septa in hyphae from the control groups; the “blue arrows” represented hyphae with wrinkled obviously at the septa after exposure to 6-methyl-2-heptanone in Figure 2D.

Because 6-methyl-2-heptanone may be a potential agent for controlling potato early blight, pure 6-methyl-2-heptanone purchased from a company was tested for antifungal activity. In detail, divided dishes were used to evaluate the inhibition of 6-methyl-2-heptanone against *A*. *solani* mycelial growth and its pathogenicity. 6-Methyl-2-heptanone suppressed mycelial growth by more than 78% at its highest dose (15 μL) (Figure 2A). For this compound, an EC_50_ value of 10.88 μL was obtained.

Mycelial structures play vital roles in the infection process, and they form a special structure, the penetration peg, before invading plant leaves. Thus, we evaluated the potential effects of 6-methyl-2-heptanone on *A*. *solani* mycelial ultrastructures. Scanning electron microscopy (SEM) was used to study the surface morphological changes of *A*. *solani*. As shown in Figure 2B, the control hyphae had smooth surfaces and intact morphology. In contrast, some 6-methyl-2-heptanone-treated hyphae had wrinkled surface cells (red arrows, Figure 2B). Hyphae exhibited swelling tissues on the surface (yellow arrows, Figure 2B) after exposure to 6-methyl-2-heptanone. Moreover, compared with normal hyphae, some of the *A. solani* 6-methyl-2-heptanone-treated mycelia were deformed and significantly enlarged (green arrows, Figure 2B).

Because the 6-methyl-2-heptanone-treated *A*. *solani* hyphae showed serious surface structure abnormalities, their internal structure used transmission electron microscopy (TEM). As shown in Figure 2C, in cross-sections, hyphae from control groups were elliptical-shaped with a clear outer cell-wall edge, cytoplasmic membrane and uniform periplasmic space. The cytoplasm was evenly distributed, with a consistent electron density. Nuclei, vacuoles, and mitochondria were also distributed randomly in the cells (Figure 2C). Compared with the undamaged control cells, a wide range of misshapen and severely deformed cells were observed in treated *A*. *solani*. Cell walls were thinner (green arrows, Figure 2C) and even damaged (red arrows, Figure 2C), which resulted in the movement of the cytoplasmic content toward the ruptured cell walls or cytoplasmic membranes (red arrow, Figure 2C). The cytoplasm was shrunken, and the number of inclusions decreased (yellow arrows, Figure 2C). Longitudinal sections of hyphae from the control groups were normal size with similar width and had clear septa (red arrows, Figure 2D). However, in the treated group, the mycelia were inflated. Many hyphae also appeared wrinkled obviously at the septa after exposure to 6-methyl-2-heptanone (blue arrows, Figure 2D). Moreover, more and larger lipid droplets were observed in the cytoplasm of treated samples compared with the control groups, which had only a few dark internal lipid droplets (Figures 2C,D).

### The Cell-Membrane Permeability of *A. solani* Hyphae Changed After the 6-Methyl-2-Heptanone Treatment

**FIGURE 3:**
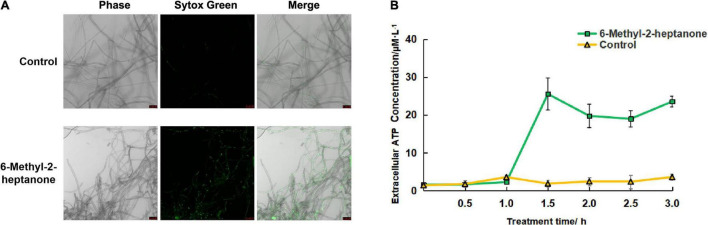
Effects of 6-methyl-2-heptanone on the cell-membrane permeability of *A. solani* hyphae. **(A)** Fluorescence microscopy imaging of *A. solani* hyphae treated with 6-methyl-2-heptanone and a control group. Membrane permeability changes in *A. solani* mycelial cells were detected using SYTOX Green dye after a 6-h incubation with an EC_50_ value of 6-methyl-2-heptanone. Green fluorescence illumination in treated samples signifies membrane damage in fungal cells. The phase channel showed all fungal cells on bright-field images, and the SYTOX Green channel showed cells attached or inserted with SYTOX Green labeled; the merge channel showed the proportion of SYTOX Green-labeled cells. **(B)** Effects of 6-methyl-2-heptanone on ATP release from *A. solani*. The changes in the extracellular ATP levels of *A. solani* represent cell-membrane damage. The ATP level was measured using an ATP kit based on a luminescent ATP assay and is presented in relative light units. The results are presented as the means ± SDs (*n* = 3).

To further identify the mechanisms by which 6-methyl-2-heptanone produces the antifungal activity against *A*. *solani* hyphal cells, we used SYTOX Green labeling to detect the membrane permeability. As shown in Figure 3A, none of the cells fluorescent green in the control group. In contrast, some cells fluorescent green in samples that had been exposed in the EC_50_ value of 6-methyl-2-heptanone for 4 days.

To further identify the damaging effects of 6-methyl-2-heptanone on cell membranes, the adenosine triphosphate (ATP) contents released into the extra-cellular medium were used as an indicator of the effects of 6-methyl-2-heptanone on the membranes of fungal hyphae. As shown in Figure 3B, the extracellular ATP level of *A. solani* rapidly increased after 1 h exposure to the EC_50_ value of 6-methyl-2-heptanone. In contrast, the extracellular ATP levels of the control cells were constantly low, from 0.5 to 3 h, which suggested that 6-methyl-2-heptanone caused serious ATP leakage from *A*. *solani* hyphal cells. Thus, the results further indicated that 6-methyl-2-heptanone increased *A*. *solani* cell-membrane permeability.

### 6-Methyl-2-Heptanone Inhibited Conidial Vitality and Damaged Internal Structures of *A. solani* Conidia

**FIGURE 4:**
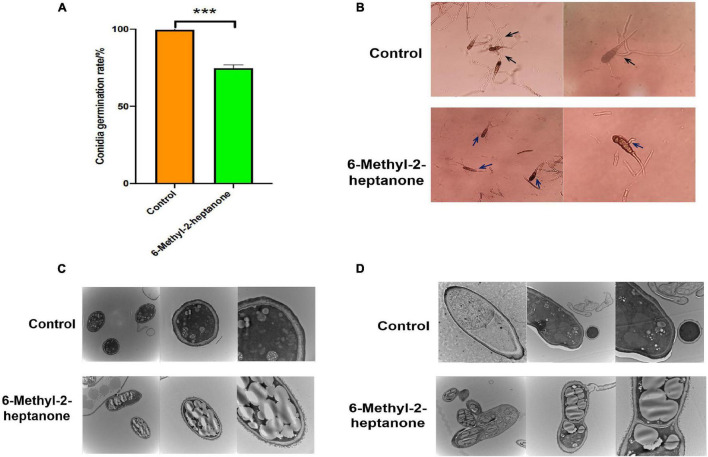
Effects of 6-methyl-2-heptanone on conidia of *A. solani*. Here, 100 μL of *A. solani* HWC-168 spore suspension (10^5^ cfu/mL) was spread onto 1.0% water agar medium on a divided dish, and the other side of the dish was inoculated with an EC_50_ value of 6-methyl-2-heptanone. **(A, B)** The conidial germination of *A. solani* in the control and 6-methyl-2-heptanone-treated groups, respectively. The “black arrow” represented conidia germinating normally with long germ tubes in the control sample; the “blue arrow” represented conidia with irregular germ tubes exposed to 6-methyl-2-heptanone. **(C, D)** Transmission electron micrographs of *A. solani* conidia in control samples and co-cultured with 6-methyl-2-heptanone samples. The results are presented as the means ± SDs (*n* = 3). *** above points represent significant differences (*p* < 0.01).

In addition to mycelia, conidial vitality also plays a crucial role during the infection of fungal pathogens. Conidia resist severe environmental conditions and are spread by wind and rain. After adhering to potato leaves, conidia form penetration pegs, special mycelial structures, which infect potato leaves. Therefore, the capacity of 6-methyl-2-heptanone to suppress conidial vitality was evaluated *in vitro*. As shown in Figure 4A, the germination rate of conidia treated with the EC_50_ value of 6-methyl-2-heptanone was 74.73% ± 2.11%, whereas it was 99.62% ± 1.67% in the control group, which suggested that 6-methyl-2-heptanone inhibited *A*. *solani* conidial germination significantly (*p* < 0.05). Conidia in the control sample germinated normally and formed long germ tubes (black arrow, Figure 4B) to infect plants. However, conidia exposed to 6-methyl-2-heptanone formed irregular germ tubes (blue arrow, Figure 4B). This shorter tube did not have the ability to penetrate and invade host epidermal cell junctions.

Transmission electron microscopy was then used to detect the degree of damage to conidial structures. The majority of *A*. *solani* conidia treated with 6-methyl-2-heptanone showed severe morphological disruptions. Collapsed shrunken were detected. Additionally, more extracellular secretions occurred around the conidial cell-wall surface, and larger lipid droplets appeared within the conidia (Figures 4C,D). In the control group not exposed to 6-methyl-2-heptanone, conidia exhibited regular shapes, uniformly distributed cytoplasm and apparently intact envelopes. We also observed electron dense cytoplasm and robust ultra-structures in the control group (Figures 4C,D). The findings confirmed that 6-methyl-2-heptanone damaged conidial structures.

### 6-Methyl-2-Heptanone Downregulated the Expression of Pathogenic Genes in *A. solani*

Because 6-methyl-2-heptanone showed strong effects on mycelial integrity and conidial structures, we further investigated the action mode of 6-methyl-2-heptanone against *A. solani* by examining the expression levels of pathogenic mycelia- and conidia-related genes. In our previous study, we determined that the *slt2* gene is involved in mycelial growth, penetration, and pathogenicity (Zhang D. et al., 2020). Moreover, fungi spread through spores, and conidia also play a key role in the *A. solani* infection process. The complete genome of *A. solani* HWC-168 has been sequenced and analyzed (Zhang et al., 2018), and one typical gene, *wetA*, related to conidia was found in the genome. Therefore, we investigated the effects of 6-methyl-2-heptanone transcription on the expression profiles of s*lt2* and *wetA* in *A. solani* using qRT-PCR.

**FIGURE 5:**
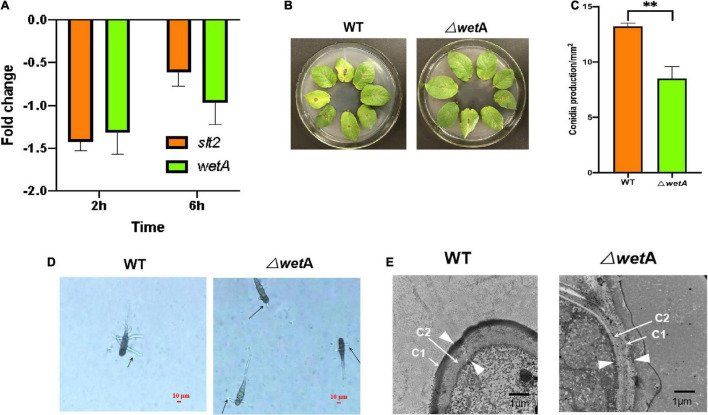
*WetA* is key conidia-related gene in *A. solani*. **(A)** Transcriptional expression profiles of *slt2* and *wetA* after co-culturing with 6-methyl-2-heptanone for 2 and 6 h. **(B)** Symptoms of early blight disease on potato leaf caused by wild-type *A. solani* (WT) and mutants (Δ*wetA*). **(C)** The sporulation yield of WT) and Δ*wetA*). **(D)** The length of Δ*wetA* mutant and WT germ tubes after a 2-h heat treatment. The “arrows” represented the lengths of Δ*wetA* mutant and WT germ tubes. **(E)** Transmission electron micrographs of conidia of WT and Δ*wetA*. The “arrows” represented C1 and C2 layers respectively; the “arrowheads” represented the width of conidial wall. The results are presented as the means ± SDs (*n* = 3). ** above points represent significant differences (*p* < 0.05).

After *A. solani* strain HWC-168 was exposed to 6-methyl-2-heptanone for 2 h and 6 h, the expression of *wetA* was strongly repressed (approximately 1.31- and 0.96-fold, respectively) (Figure 5A). The transcriptional expression of *slt2* was induced (approximately 1.42-fold) compared with the control group and then repressed (approximately 0.61-fold) in the presence of VOCs after 2- and 6-h co-culturing (Figure 5A). The down-regulated expression levels of *slt2* and *wetA* were consistent with the virulence reduction in *A. solani*.

### *WetA* Is a Conidia-Associated Gene in *A. solani*

Identifying the functions of pathogenic genes is important for determining pathogenic mechanisms and biological control approaches for *A. solani*. Moreover, little is known about the functions of *A. solani* pathogenesis-related genes. Because 6-methyl-2-heptanone showed a strong effect on the transcriptional expression of *wetA* in conidia, we compared the *wetA* gene sequence in *A. solani* with those of 22 fungi. The *wetA* gene in *A. solani* is closely related to the *wetA* gene in *Alternaria alternate* (Supplementary Figure 1). Then, the functions of *wetA* were determined using gene knockouts and phenotype verification.

To determine whether *wetA* affects the pathogenicity of *A. solani*, the virulence of the deletion mutants and the wild-type (WT) strain were compared *in vivo* using potato leaf tests. As shown in Figure 5B, potato leaves inoculated with WT HWC-168 showed obvious lesions and yellow halos. The lesion diameters extended to 0.50 ± 0.07 cm after a 7-day incubation at 25°C, whereas for the leaves inoculated with Δ*wetA* mutants, the lesion diameters were limited to 0.20 ± 0.06 cm. Thus, the deletion of *wetA* significantly reduced the pathogenicity of *A. solani* (*p <* 0.05).

Then, we evaluated the sporulation and conidia germination of deletion mutants and the WT strain under *in vitro* conditions. As shown in Figure 5C, the sporulation yield of the *wetA* deletion mutant per area was 8.51 ± 1.09 × 10^2^/mm^2^, whereas that of the WT strain was 13.21 ± 0.28 × 10^2^/mm^2^. Compared with the WT strain, the sporulation yield of the *wetA* deletion mutant per area decreased significantly (*p* < 0.05). These results suggested that *wetA* has a significant role in sporulation. Moreover, we examined the conidial germination of WT *A. solani* and mutants (Δ*wetA*) after a 2-h heat treatment. The conidial germination of WT was 40.67% ± 2.52%, whereas that of the Δ*wetA* mutants was limited to 27.33% ± 1.17%. The lengths of Δ*wetA* mutant and WT germ tubes were 18.56 ± 2.89 μm and 57.96 ± 4.90 μm, respectively (Figure 5D). The results indicated that expression of the *wetA* gene had strong inhibitory effects on conidial germination and germ tube elongation under heat-treatment conditions.

To examine the role of *wetA* in conidial vitality, WT and *wetA* mutant conidia in *A. solani* were compared using TEM. As shown in Figure 5E, WT conidia formed a crenulated electron-dense C1 layer and a condensed electron-light C2 layer. In the *wetA* mutants, although the C1 and C2 layers were formed, the C1 layer was not crenulated and the C2 layer failed to condense, resulting in a thicker conidial wall than in the WT strain. Moreover, the C1 layer was subtended by projections from the C2 layer in the WT strain, and this was not observed in the *wetA* mutant strain. These data indicated that the *wetA* mutant exhibits conidial wall defects similar to those found in *Aspergillus nidulans* (Sewall et al., 1990; Marshall and Timberlake, 1991) and that *wetA* plays an essential role in conidial wall completion and spore maturation.

## Discussion

Mycelial growth, hyphal morphology, and conidial germination are significant factors in the plant infection processes of fungal pathogens. Consequently, most previous studies focused on plant fungal mycelial morphology and spore germination after being treated with VOC mixtures emitted by bacterial strains (Chaurasia et al., 2005; Effmert et al., 2012; Morath et al., 2012; Zheng et al., 2013; Chantal et al., 2014; Xie et al., 2018; Li et al., 2019; Martins et al., 2019; Wu et al., 2019; Tran et al., 2020). For example, the VOCs of *Bacillus velezensis* 5YN8 suppress the mycelial growth and conidial formation of *B*. *cinerea* BC1301 (Jiang et al., 2018). Excessive vesication or thickened cell walls in conidia and increased plasma membrane retractions have been observed by TEM in mycelia of *B*. *cinerea* fumigated with *Bacillus* VOCs (Li et al., 2012). In our previous study, we also found that volatiles secreted by the ZD01 strain inhibit mycelial growth and conidial germination (Zhang D. et al., 2020). Thus, most of studies have focused on the effects of volatile mixtures produced by *Bacillus* stains against plant fungi. However, the action sites of different component(s) of the volatile mixtures may vary in different fungi, and little is known about the action mechanisms of specific effective substances in VOCs on plant pathogens.

In our study, 6-methyl-2-heptanone accounted for relatively large contents, at 22.27 and 8.88% of the total VOCs from *B. subtilis* ZD01, using FFAP and HP-5 capillary columns, respectively, in GC-MS analyses, and it inhibited *A. solani* mycelial growth strongly *in vitro* (Zhang D. et al., 2020). Furthermore, 6-methyl-2-heptanone is produced by *Bacillus* strains and shows significant antifungal activities against plant pathogens. For example, 6-methyl-2-heptanone produced by *Bacillus vallismortis* 12a and *Bacillus altitudinis* 14b completely inhibits the mycelial growth of *Monilinia fructicola* (Liu et al., 2018). Therefore, 6-methyl-2-heptanone may be a key active chemical component of VOCs emitted from *Bacillus* strains that can be used for controlling plant disease. Consequently, we selected it as a specific effective substance to elucidate the action mechanisms against *A. solani*. We found that 6-methyl-2-heptanone damaged cell-wall integrity and changed cell-membrane permeability. Cell walls and membranes are crucial for maintaining cell viability (Bowman and Free, 2006; Ruiz-Herrera et al., 2006; Shao et al., 2013; Tao et al., 2014). It is, therefore, necessary to reveal the interactions of bioactive VOCs with model membranes.

Currently, little is known about the functions of *A. solani* pathogenesis-related genes, which may be important for the biological control of plant pathogens. In this study, the function of the *wetA* gene, which is involved in conidial vitality in *A. solani*, was identified using a constructed knockout mutant and phenotypical characterization. The *wetA* mutant strain failed to form condensed C1 and C2 layers, which was consistent with previous studies (Sewall et al., 1990; Marshall and Timberlake, 1991). In *A. nidulans*, the *wetA* gene is required late in development for the synthesis of crucial cell-wall layers (Marshall and Timberlake, 1991). The inner wall layer of *wetA* mutant conidia did not condense during Stage II, and they form large cytoplasmic vacuoles that undergo lysis (Sewall et al., 1990). Here, the WT C1 layer was slightly more crenulated than that of the Δ*wetA* strain. The C2 layer appears condensed in the WT but not in the *wetA* mutant strain (Sewall et al., 1990). Therefore, *wetA* plays crucial roles in the sporulation and conidial wall formation of *A. solani*, which had further effects on its pathogenicity in *in vivo* tests.

**FIGURE 6:**
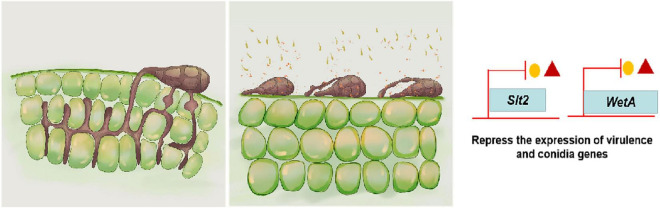
A model for the mode of action of 6-methyl-2-heptanone produced by ZD01 against *A. solani*. 6-Methyl-2-heptanone changes the structures of mycelia and conidia of *A. solani*, which subsequently leads to the suppression of fungal growth, mycelium penetration, conidia vitality, and germination, as well as relative virulence and conidia-related gene expression levels. As a result, *A. solani* failed to infect potato leaves.

In summary, this study first elucidated the action mechanism of *B*. *subtilis* ZD01 metabolite 6-methyl-2-heptanone to control *A*. *solani* and shed light on the potential biocontrol mechanism of 6-methyl-2-heptanone against *A. solani* in potato (Figure 6). 6-Methyl-2-heptanone caused hyphal deformity and damaged the cell integrity and membrane permeability of *A. solani* hyphae, which could not form the penetration pegs. Additionally, it inhibited conidial germination and altered conidial structures. Moreover, 6-methyl-2-heptanone down-regulated the transcriptional expression levels of *slt2* and *wetA* genes, which are involved in mycelial vitality, sporulation, and conidial maturity. Future research will focus on increasing the safety of 6-methyl-2-heptanone treatments and determining the action sites of 6-methyl-2-heptanone produced by *B*. *subtilis* ZD01 against *A*. *solani*.

## Experimental Procedures

### Optimization of Volatile Organic Compound Collection Conditions

Here, 20 μL of *Bacillus* strain suspension (1 × 10^8^ cfu/mL) was inoculated into 6 mL LB medium in a 20-mL headspace-vial. The vials were firmly sealed using parafilm and rubber lids, and then, they were incubated at 25°C for 4 days before VOC collection. HS-SPME and GC-MS were used to analyze the samples.

A SPME holder from Supelco, Inc. (Bellefonte, PA, United States) was used to perform HS-SPME manually. The SPME fibers were also purchased from Supelco, Inc. The extraction conditions were optimized in accordance with a previous method (Zhang et al., 2021). Briefly, the vials were water-bathed in a heated metal block with the SPME fiber inserted in the headspaces under different extraction conditions for optimization. We used the extraction fiber, temperature, and time as factors. We chose 85-μm Polyacrylate, 75-μm Carboxen/PDMS, and 50/30-μm DVB/CAR/PDMS as the extraction fibers for optimization at 40°C condition for 40 min. Next, the extraction temperature was optimized using the optimal fiber for 40 min at 30, 40, 50, and 60°C. To optimize the extraction time, the VOCs were extracted for 20, 30, 40, and 50 min with the optimized fiber and temperature. Finally, the VOCs were extracted using the optimized conditions. The experiment was repeated three times. All the strains used in this study are listed in Supplementary Table 1.

### Analysis of Volatile Organic Compounds From *B. subtilis* ZD01 by Gas Chromatography-Mass Spectrometry

After extraction, the analytes were desorbed for 5 min at 250°C in the injector of the GC with the purge valve off (split-less mode). Capillary GC-MS was carried out using a Thermo Trace 1310 gas chromatographer (Thermo Fisher Scientific, Waltham, MA, United States). The samples were analyzed on a FFAP capillary column (60 m × 0.25 mm × 0.25 μm, Thermo Fisher Scientific). The temperature of the injection port was 230°C. The flow rate was 1 mL/min. We used the following temperature program: start at 50°C; increase to 220°C at 5°C/min; and hold at 220°C for 15 min.

A Thermo TSQ-8000 MS was used for peak separation and detection. The MS was operated in electron ionization mode at 70 eV with a source temperature of 280°C using a continuous scan from 45 *m/z* to 450 *m/z*. The ionization source and transfer line temperatures were both 230°C, and electron ionization mode was used. The analysis was performed in full-scan mode. Mass spectral data for the volatile compounds were compared with data in the National Institute of Standards and Technology (NIST) Mass Spectrum Library. The VOCs in treated samples that were not found in the control were considered the final *Bacillus*-produced VOCs. The experiment was repeated three times.

For each detected peak, a standard mixture of hydrocarbons from C_7_ to C_27_ (Bailingwei Inc., Beijing, China) was prepared. The sample and the hydrocarbon standard mixture were co-injected into the GC, and the retention times were used to calculate retention indices. A linear retention index was calculated using GC retention index standards in accordance with the method of Van den Dool and Kratz (1963). The relative strength indexes of chemicals that matched chemicals in the NIST library with scores greater than 800 were used.

### Inhibition Assay of 6-Methyl-2-Heptanone Against *A. solani in vitro*

To test the inhibition on mycelial growth, pure 6-methyl-2-heptanone was purchased from Shanghai Macklin Biochemical Technology Co., Ltd. In this test, the divided plate method was used (Xie et al., 2018), and the plate allocation of the different treatments was randomized. Briefly, a 5-mm square plug from an *A*. *solani* mycelial agar disk was placed in one compartment of the divided plate containing PDA medium, and the other compartment containing PDA medium was incubated with different aliquots of pure compound. The doses of 6-methyl-2-heptanone were 3, 6, 9, 12, and 15 μL, respectively. The dishes were immediately sealed with parafilm and incubated at 25°C for 4 days. Pathogens and plates not containing the pure compound were used as controls. The sample unit was represented by six replicates per dose. The inhibitory rate on mycelial growth was calculated in accordance with the following formula, and EC_50_ values were calculated as the effective concentrations that inhibited fungal mycelial growth by 50% in comparison with the control.

Inhibitory rate on mycelium growth (%) = (the diameter of control – the diameter of treatment group)/the diameter of control × 100%.

The efficiency of 6-methyl-2-heptanone against *A*. *solani* conidial germination was also tested using the divided plate assay. For this purpose, 100 μL of *A*. *solani* HWC-168 spore suspension (10^5^ cfu/mL) was spread onto half of the 1.0% water agar medium in a divided dish, and the other side of dish was inoculated with the EC_50_ value of 6-methyl-2-heptanone. The spore suspension and LB plates with sterile water were used as controls. Dishes were immediately sealed with parafilm. The plates were incubated at 25°C for 6–8 h, and sporulation was assessed. The experiment was repeated in triplicate.

Inhibitory rate on conidial germination (%) = (the conidial germination of control – the conidial germination of treatment group)/the conidial germination of control × 100%.

### Scanning Electron Microscopy

Scanning electron microscopy was conducted to determine the effects of 6-methyl-2-heptanone on the hyphae of *A*. *solani* at the ultra-structural level. The *A*. *solani* mycelia were inoculated as described in the above divided plate assay and cultured with EC_50_ doses of 6-methyl-2-heptanone at 25°C for 4 days. The plates without 6-methyl-2-heptanone were used as controls. Then, mycelia of each group were harvested and fixed in 2% glutaraldehyde (Solarbio, Beijing, China) at 4°C and dehydrated with gradient ethanol solutions (50, 70, 80, 90, and 100%) for 20 min. Afterward, ethanol was replaced by 100% tertiary butyl ethanol. Cells were then freeze-dried, coated with gold, and imaged using a Hitachi S-3500N field emission SEM (Hitachi, Tokyo, Japan).

### Transmission Electron Microscopy

Transmission electron microscopy was used to observe internal morphological changes in *A*. *solani* colonies. For groups exposed to pure 6-methyl-2-heptanone, the *A*. *solani* mycelia and conidia were treated with E_*C50*_ doses of 6-methyl-2-heptanone as described in the above divided plate assay. The plates without 6-methyl-2-heptanone were used as controls. Then, hyphae and conidia were collected. For fungal deletion and WT strains, conidia were collected. The conidia were collected by centrifugation (5,000 × *g* for 15 min at 20°C). Hyphae and conidia were washed and fixed with 2% glutaraldehyde for 30 min at 4°C. The specimens were prepared in accordance with Yamanaka et al. (2005) for TEM analysis. Ultra-structural changes in the cells were observed using a Hitachi H-7650 transmission electron microscope (Hitachi).

### Extracellular Adenosine Triphosphate Measurement Assay

The same design as described above was used to investigate the effects of 6-methyl-2-heptanone on outer mycelial ATP contents. For A. *solani*, a 5-mm square plug of mycelial agar disks was placed in one compartment of the divided plate containing PDA medium, and the plate was incubated at 25°C for 4 days. Then, an EC_50_ dose of 6-methyl-2-heptanone was added to the other compartment and incubated for 0.5, 1.0, 1.5, 2.0, 2.5, and 3.0 h at 25°C. The plates without 6-methyl-2-heptanone were used as controls. The *A*. *solani* cells and the supernatants were collected by centrifugation (12,000 × *g* for 5 min at 4°C). The extracellular ATP level was determined using an Enhanced ATP Assay Kit (Beyotime Biotechnology Inc., Shanghai, China) and a multi-function microplate reader (Tecan Spark, Salzburg, Austria). The ATP kit was based on a luminescent ATP assay protocol that involved the lysis of each cell sample and the addition of luciferase and luciferin, followed by the measurement of the emitted light. The experiment was repeated in triplicate.

### Fluorescence Microscopy Imaging

For A. *solani*, a 5-mm square plug of a mycelial agar disk was placed in one compartment of a divided plate containing PDA medium, and the plate was incubated at 25°C for 4 days. One section of the cells was treated with an EC_50_ dose of 6-methyl-2-heptanone for 6 h at 25°C, and the other section was treated with sterile saline for 6 h at 25°C. The mycelia were collected and re-suspended in sterile saline. Then, 0.8 μM SYTOX Green solution (Invitrogen Corporation, Carlsbad, CA, United States) was added to all the cells. The samples were incubated for 15 min in the dark. Afterward, mycelia were rinsed two times with 8.5% sterile saline, re-suspended in sterile saline, and immediately measured for fluorescence. Green fluorescent signaling in *A*. *solani* was visualized using a Nikon Ti2-U fluorescence microscope (Nikon Corporation, Tokyo, Japan). The excitation wavelength was 488 nm, and the emission wavelength was 538 nm (Mu Oz et al., 2013).

### Quantitative Real-Time PCR

Total RNAs of *A. solani* cells co-cultured with 6-methyl-2-heptanone for 2 and 6 h were extracted using a Bacterial RNA Kit (Omega Bio-Tek, Norcross, GA, United States) in accordance with the manufacturer’s instructions. First-strand cDNA was obtained using reverse transcriptase (TransGen Biotech, Beijing, China) with random hexamer primers. Real-time PCR was performed with SYBR Premix Ex Taq™ (TransGen Biotech). The actin gene was used as an internal reference gene. The specific primers used are listed in Supplementary Table 2. The relative expression levels of specific genes were calculated using the 2^–ΔΔ*CT*^ method (Livak and Schmittgen, 2001).

### Construction of Fungal Deletion Strains

Gene deletion vector construction and the transformation of *A. solani* were performed using the double-joint PCR method with minor modifications (Yu et al., 2004). The primers used for flanking sequence amplification for each gene are listed in Supplementary Table 2. A hygromycin resistance cassette replaced the open reading frame of *wetA*, and the constructed fragment was inserted into the pEASY-T1 cloning vector (Supplementary Table 1). After transforming the constructed plasmid into HWC-168, the subsequent deletion mutants were verified by PCR using the *wetA*-F/R primer set (Supplementary Table 2).

### *In vivo* Antagonistic Activity of *WetA* Mutants

To determine the pathogenicity of different *A. solani* strains, 20 μL of conidial suspensions (10^5^ cfu/mL) of WT and *wetA* mutants were inoculated onto the center of one piece of fresh potato leaf. After 5 days of growth under a 12-h/12-h light/dark cycle at 25°C, the lesion diameters were measured.

### Statistical Analysis

Three independent experiments were performed for each assay. Data were analyzed using SPSS 20.0 Windows Software (SPSS Inc., Chicago, IL, United States). Least significant differences were calculated to compare results at the 0.05 level.

## Data Availability Statement

The datasets presented in this study can be found in online repositories. The names of the repository/repositories and accession number(s) can be found below: https://www.ncbi.nlm.nih.gov/genbank/, CP046448.1.

## Author Contributions

DZ, RQ, and YF performed the experiments. DZ wrote the manuscript. JZ and JC provided data curation and methodology. DMZ provided technical assistance. DZ, ZY, and JHZ designed the experiments. ZY and JHZ provided supervision and project administration. All authors contributed to the article and approved the submitted version.

## Conflict of Interest

The authors declare that the research was conducted in the absence of any commercial or financial relationships that could be construed as a potential conflict of interest.

## Publisher’s Note

All claims expressed in this article are solely those of the authors and do not necessarily represent those of their affiliated organizations, or those of the publisher, the editors and the reviewers. Any product that may be evaluated in this article, or claim that may be made by its manufacturer, is not guaranteed or endorsed by the publisher.
