# Supplementary material for: Mechanism of a Volatile Organic Compound (6-Methyl-2-Heptanone) Emitted From Bacillus subtilis ZD01 Against Alternaria solani in Potato
Source: Front Microbiol. 2022 Jan 13;12:808337. doi: 10.3389/fmicb.2021.808337 (PMC8793485; doi:10.3389/fmicb.2021.808337)
Supplement: Supplementary file 1 [file Data_Sheet_1.doc]

**Table S1 Strains and plasmids used in this study**

| **Strain and plasmid** | **Description** | **Source** |
| --- | --- | --- |
| **Strains** | | |
| *Bacillus subtilis* ZD01 | Undomesticated environmental strain | Lab strain collection |
| *Alternaria solani* HWC-168 | Undomesticated environmental strain | (Zhang, He et al. 2018) |
| HWC-168-A | Δ*wetA*::hyg in HWC-168 (knock-out) | This study |
| **Plasmid** | | |
| pEASY-T1 | Cloning vector | TransGen Biotech |

**Table S2 Oligonucleotides used in this study**

| **Primer** | **Description** | **Sequence(5' →3' )** |
| --- | --- | --- |
| slt2-RT-F | *slt*2 qRT-PCR | CACTATTCAGGAACGCCAACC |
| slt2-RT-R | *slt*2 qRT-PCR | GACGACCTCAAACTGGAAATCAA |
| wetA-F | *wetA* amplification and deletion verification | ATGCCAATCCGACCTGCCAG |
| wetA-R | *wetA* amplification and deletion verification | TTAACCCTCCATTACCAGTA |
| wetA-RT-F | *wetA* qRT-PCR | CATCATCATCATCACACACAAC |
| wetA-RT-R | *wetA* qRT-PCR | TCACAAAACCAACGCCAC |
| wetA-UP-F | *wetA* upstream fragment amplification | TCAATCTACTCCTGGACCCGAACAAG |
| wetA-UP-R | *wetA* upstream fragment amplification | GCCCAAAAATGCTCCTTCAAACGTGCCAATGATGCCTCATAACA |
| wetA-DOWN-F | *wetA* downstream fragment amplification | CCCTGGGTTCGCAAAGATAATGGGGCCATTGTTTTACATATACGA |
| wetA-DOWN-R | *wetA* downstream fragment amplification | GGTGGGAACAAAGAAGTGGAACG |
| hyg-F | Amplify hygromycin resistance cassette | TTGAAGGAGCATTTTTGGGC |
| hyg-R | Amplify hygromycin resistance cassette | TTATCTTTGCGAACCCAGGG |
| actin-F | Actin gene qRT-PCR | AGTCTTCCCTTCCATCGTCG |
| actin-R | Actin gene qRT-PCR | CTTCTCCATGTCGTCCCAGT |

**REFERENCES**

Zhang, D., et al. (2018). "Genome sequence of the potato pathogenic fungus Alternaria solani HWC-168 reveals clues for its conidiation and virulence." **18**(1): 1-13.


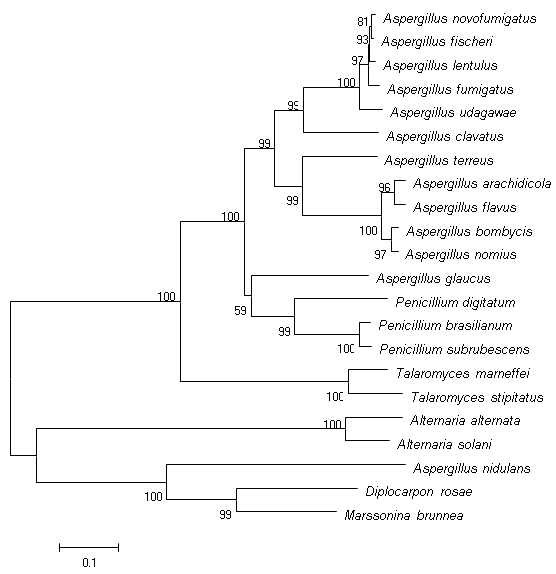


Fig S1. UPGMA tree constructed for different *wetA* genes in various fungi
